# Hemophagocytic Lymphohistiocytosis Secondary to Extrapulmonary Tuberculosis in an HIV Patient: A Case Report

**DOI:** 10.7759/cureus.101529

**Published:** 2026-01-14

**Authors:** Elton Anselmo Junior

**Affiliations:** 1 Internal Medicine, Universidade Estadual de Campinas, Campinas, BRA

**Keywords:** endemic infections, hemophagocytic lymphohistiocytosis (hlh), hiv aids, immunocompromised patient, : tuberculosis

## Abstract

Hemophagocytic lymphohistiocytosis (HLH) is a severe hyperinflammatory syndrome caused by unregulated immune activation, leading to a "cytokine storm" and multisystem failure. In people living with HIV (PLWH), it is frequently triggered by opportunistic infections like *Mycobacterium tuberculosis*. This report describes a 28-year-old transgender woman with HIV (on ART, undetectable viral load) admitted with a 20-day history of high fever, diarrhea, and hepatosplenomegaly. Admission labs showed severe pancytopenia (Hb 6.2 g/dL, WBC 1,590/µL, platelets 102,000/µL) and a CD4 count of 164 cells/mm³. Investigation revealed extreme hyperferritinemia (peaking at 18,366 ng/mL) and a positive urinary lateral flow urine lipoarabinomannan assay (LF-LAM) test, indicating extrapulmonary tuberculosis (TB). Abdominal CT showed mesenteric and para-aortic lymphadenopathy. Bone marrow biopsy confirmed HLH through frequent hemophagocytosis figures. Despite immediate initiation of antituberculosis therapy (ATT) combined with intravenous immunoglobulin, methylprednisolone pulse, and cyclosporine, the patient developed acute kidney injury and severe hepatitis, progressing to refractory shock and death on the 37th day. This case highlights the diagnostic challenge of HLH due to clinical overlap with sepsis. The LF-LAM test was crucial for rapid TB identification. Although integrated immunomodulation and ATT are recommended, the prognosis remains poor in advanced immunosuppression. High clinical suspicion is vital for early intervention in HIV-positive patients with fever and cytopenias in TB-endemic regions.

## Introduction

Hemophagocytic lymphohistiocytosis (HLH) is a rare and potentially fatal hyperinflammatory syndrome resulting from dysregulated immune activation, characterized by persistent proliferation of activated T lymphocytes and macrophages, with exacerbated release of pro-inflammatory cytokines [1-3]. This uncontrolled immune response leads to multisystem dysfunction, which can rapidly progress to organ failure. Clinically, HLH manifests with persistent fever, hepatosplenomegaly, cytopenias, hyperferritinemia, hypertriglyceridemia, liver dysfunction, and histological evidence of hemophagocytosis in tissues such as bone marrow, liver, and lymph nodes. The disease is classified into two forms: primary (familial) and secondary (acquired). The primary form is of genetic origin and more prevalent in children, whereas the secondary form can occur at any age and is usually associated with malignancies, infections, and autoimmune diseases. Among infectious causes, viral infections predominate (particularly Epstein-Barr and herpesvirus), followed by bacterial infections, with tuberculosis being a prominent example [1]. HLH is rare in the general population, with an estimated incidence of 1 to 2 cases/million/year. In people living with HIV (PLHIV), this incidence can be up to 36 times higher [2]. Mortality remains high, ranging from 20% to 88%, depending on the etiology, severity of the condition, prompt diagnosis, and therapeutic response. In the HIV-associated form, the rate can exceed 50%, especially in patients with CD4 lymphocyte counts < 200 cells/mm³ [3]. Given the risk of rapid progression and high lethality, early recognition and immediate institution of immunosuppressive therapy are fundamental. Therefore, this case report is justified by offering a detailed study of secondary HLH due to tuberculosis in an HIV-positive patient, illuminating the diagnostic, clinical, and therapeutic challenges. It is hoped that the discussion of this case will contribute to increased clinical surveillance, facilitate early diagnosis, and encourage more effective therapeutic interventions, with the potential to reduce mortality.

## Case presentation

A 28-year-old transgender woman, living with human immunodeficiency virus (HIV) since age 25, on regular antiretroviral therapy (ART) with an undetectable viral load, was admitted to the hospital with a 20-day history of daily fever (39-41°C), predominantly in the morning, associated with watery diarrhea, asthenia, diffuse abdominal pain, and poor appetite.

Upon physical examination, she presented with mucocutaneous pallor and hepatosplenomegaly. At admission, the complete blood count (CBC) revealed pancytopenia: hemoglobin (6.2 g/dL), white blood cell counts (1,590 cells/µL), and platelets (102,000 cells/µL).

Complementary diagnostic workup showed negative serology for hepatitis B, hepatitis C, and syphilis; IgG was positive for cytomegalovirus (CMV), Epstein-Barr virus (EBV), and toxoplasmosis. Tests including urine culture, blood cultures, cerebrospinal fluid (CSF) culture, stool ova and parasites, fibrinogen, antinuclear antibody (ANA), anti-DNA, reticulocyte count, stool culture, creatinine, urea, transaminases, and serum protein electrophoresis showed no significant abnormalities. The CD4+ T-lymphocyte count was 164 cells/mm³, and the HIV viral load remained undetectable. These tests have been summarized in Table 1.

**Table 1:** Admission exams HbsAg: Hepatitis B surface antigen; Anti-HBC: Antibody to hepatitis B core antigen; Anti-HIV 1 and 2: Anti-Retroviral Therapy; VDRL: Venereal Disease Research Laboratory; CMV: Cytomegalovirus; EBV: Epstein-Barr Virus; AST: Aspartate Aminotransferase; ALT: Alanine Aminotransferase; LF-LAM: Lateral Flow Lipoarabinomannan assay.

Exams	Admission exams	Reference value
Hemoglobin (g/dL)	6,2	12-16
White blood cells (cells/µL)	1.590	4.000-13.000
Platelets (cells/µL)	102.000	150.000-450.000
HbsAg	Negative	Negative
Anti-HBC	Negative	Negative
Anti-HIV 1 and 2	Negative	Negative
VDRL	Negative	Negative
CMV	IgM Negative / IgG Positive	Negative / Negative
EBV	IgM Negative / IgG Positive	Negative / Negative
Creatinine (mg/dL)	1,02	< 1,2
Urea (mg/dL)	41	> 50
Antinuclear Antibody	Negative	Negative
AST (U/L)	14	< 35
ALT (U/L)	20	< 35
Total bilirubin (mg/dL)	1,7	< 1,2
Ferritin (ng/mL)	> 2.000	13-150
Triglycerides (mg/dL)	196	< 150
Urinary LF-LAM	Positive	Negative
LT CD4 (céls/mm³)	164	> 350
Coproculture	Negative	Negative
Blood Culture	Negative	Negative
Fibrinogen (mg/dL)	689	150-450

**Figure 1:**
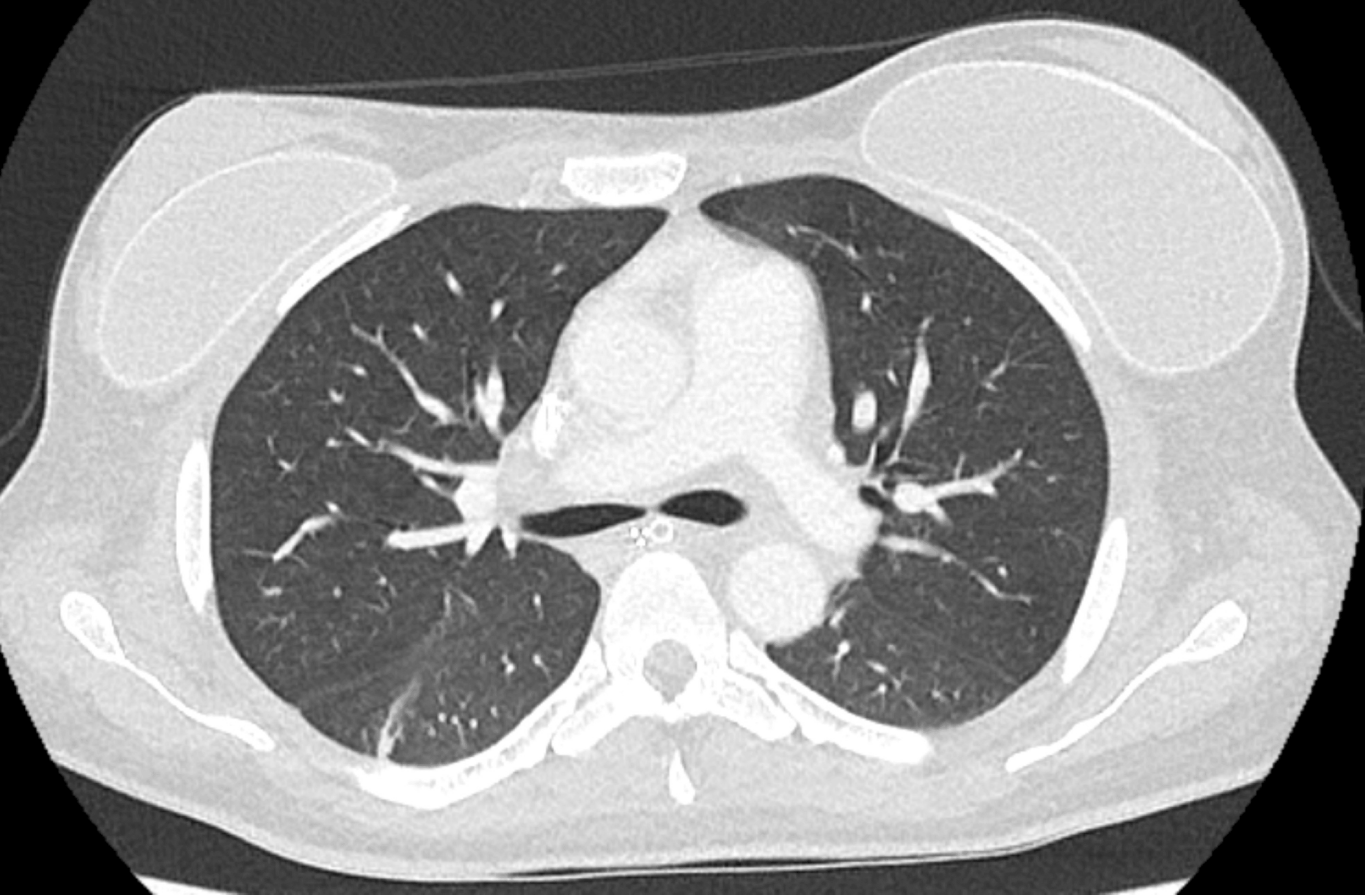
A chest CT scan showed no abnormalities.

**Figure 2:**
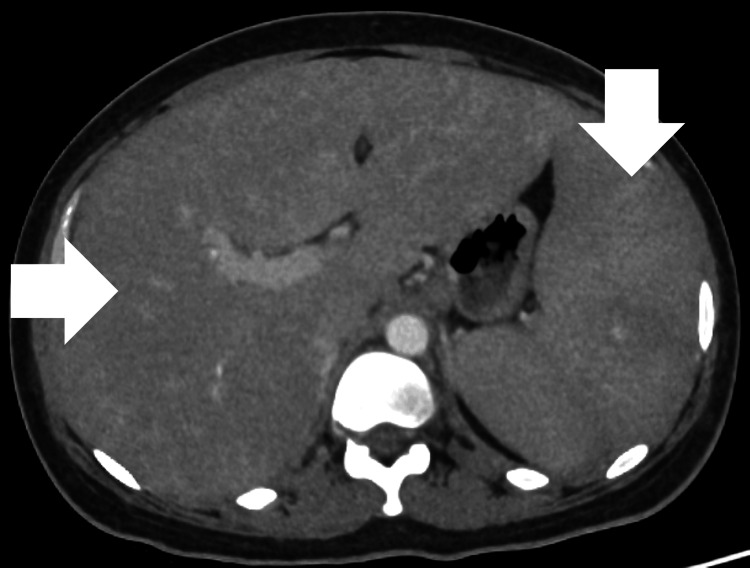
Abdominal CT scan showing hepatosplenomegaly and no other abnormalities.

Other laboratory tests showed total bilirubin of 1.7 mg/dL, ferritin > 2,000 ng/mL, triglycerides of 196 mg/dL, and a positive urinary LF-LAM test. Upper gastrointestinal endoscopy and colonoscopy were normal. Chest computed tomography (CT) showed no abnormalities (Figure 1); abdominal CT revealed hepatosplenomegaly and mesenteric and para-aortic lymphadenopathy (Figure 2).

**Figure 3:**
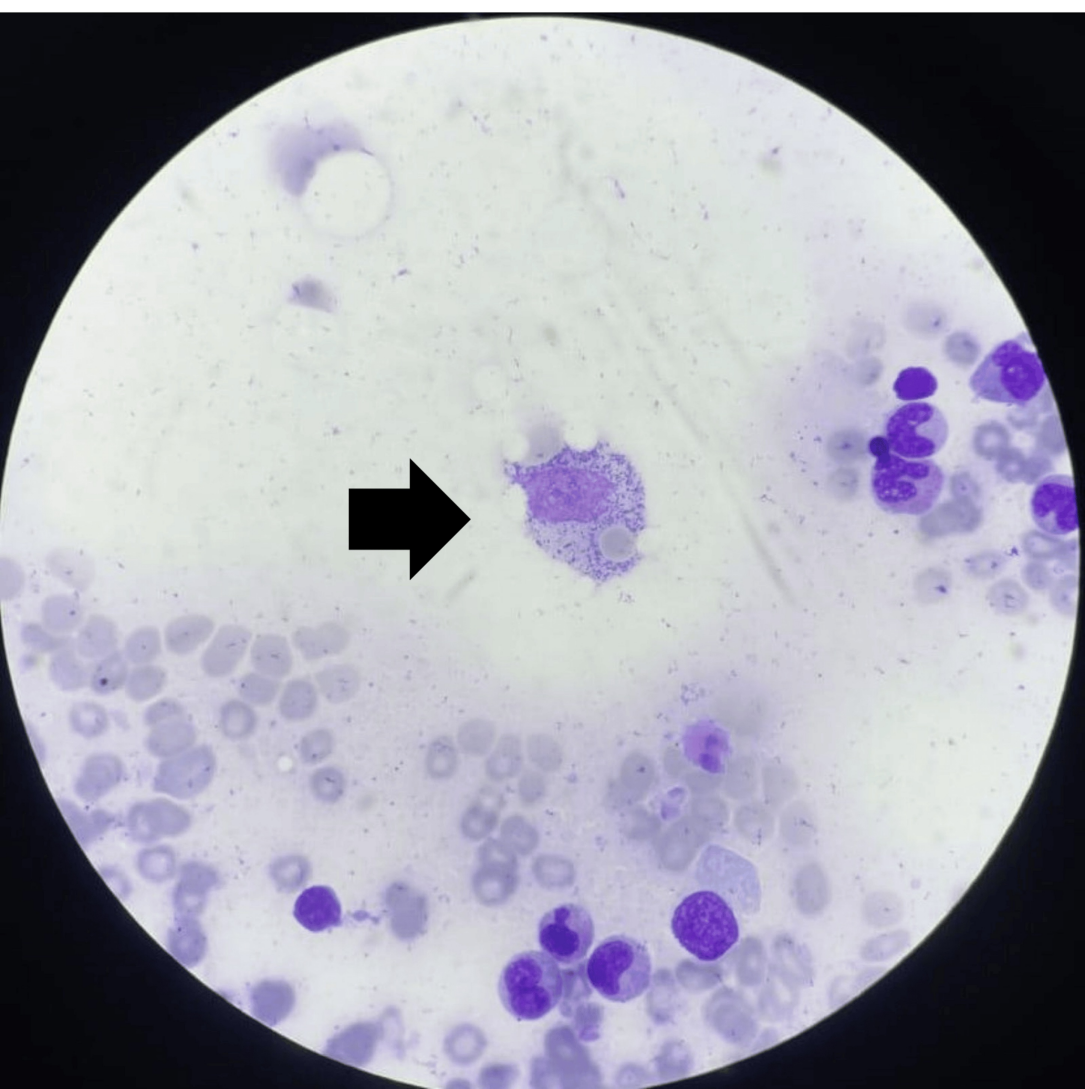
Bone marrow biopsy showing signs of hemophagocytosis.

Given the positive LF-LAM, abdominal lymphadenopathy, and diarrhea, a diagnostic hypothesis of intestinal tuberculosis was raised, and treatment with rifampin, isoniazid, pyrazinamide, and ethambutol was initiated. Simultaneously, considering the association of pancytopenia, hyperferritinemia, hepatosplenomegaly, and lymphadenopathy, the hypothesis of HLH (hemophagocytic lymphohistiocytosis) was considered. A bone marrow biopsy was performed, which reported: Bone marrow with all three hematopoietic lineages well-represented, absence of parasites, absence of immature cells, and presence of macrophages with frequent hemophagocytosis figures (Figure 3).

Following the diagnosis, intravenous immunoglobulin, pulse therapy with methylprednisolone, and maintenance with cyclosporine were instituted. Despite these measures, on the 20th day, the patient experienced clinical and laboratory deterioration, developing acute kidney injury (creatinine 3.92 mg/dL; urea 124 mg/dL), hepatitis (AST 249 U/L, ALT 330 U/L, total bilirubin 16 mg/dL), and marked hyperferritinemia (18,366 ng/mL). These and other tests from day 20 are summarized in Table 2.

**Table 2:** Labs from hospital day 20, showing the patient's clinical course. HbsAg: Hepatitis B surface antigen; Anti-HBC: Antibody to hepatitis B core antigen; Anti-HIV: Anti-Retroviral Therapy; VDRL: Venereal Disease Research Laboratory; CMV: Cytomegalovirus; EBV: Epstein-Barr Virus; AST: Aspartate Aminotransferase; ALT: Alanine Aminotransferase; LF-LAM: Lateral Flow Lipoarabinomannan assay.

Exams	Admission exams	Tests performed on the 20th day of hospitalization	Reference value
Hemoglobin (g/dL)	6,5	6,6	12-16
White blood cells (cells/µL)	1.590	1.341	4.000-13.000
Platelets (cells/µL)	102.000	122.000	150.000-450.000
HbsAg	Negative		Negative
Anti-HBC	Negative		Negative
Anti-HIV 1 and 2	Negative		Negative
VDRL	Negative		Negative
CMV	IgM Negative / IgG Positive		Negative / Negative
EBV	IgM Negative / IgG Positive		Negative / Negative
Creatinine (mg/dL)	1,02	3,92	< 1,2
Urea (mg/dL)	41	134	> 50
Antinuclear Antibody	Negative		Negative
AST (U/L)	14	249	< 35
ALT (U/L)	20	330	< 35
Total bilirubin (mg/dL)	1,7	16	< 1,2
Ferritin (ng/mL)	> 2.000	18.366	13-150
Triglycerides (mg/dL)	196	496	< 150
Urinary LF-LAM	Positive		Negative
LT CD4 (céls/mm³)	164		> 350
Coproculture	Negative		Negative
Blood Culture	Negative		Negative
Fibrinogen (mg/dL)	689	121	150-450

On the 30th day of hospitalization, the patient presented with significant psychomotor agitation followed by coma, requiring endotracheal intubation and mechanical ventilation. On the 37th day of hospitalization, the patient progressed to refractory shock and died.

## Discussion

Hemophagocytic lymphohistiocytosis (HLH) is a hyperinflammatory syndrome characterized by the unregulated activation of T lymphocytes and macrophages, leading to an intense release of inflammatory cytokines, the so-called 'cytokine storm' [4]. This state culminates in the subsequent destruction of hematopoiesis and multisystem organ failure.

The diagnosis is challenging because the clinical signs, such as fever, asthenia, abdominal pain, hepatosplenomegaly, and pancytopenia, are nonspecific and can be confused with severe sepsis, leishmaniasis, lymphomas, or autoimmune diseases [2]. This symptomatic overlap with other conditions often causes significant diagnostic delays.

The mortality rate of HLH is high, ranging between 40% and 50% even with appropriate treatment [5], and can reach even higher rates in people living with HIV (PLWH)-estimated above 40% in some studies. In the case described here, the patient had a CD4 count of 164 cells/mm³, an indicator of severe immunosuppression and a poor prognostic factor.

Diagnosis is based on the HLH-2004 criteria, which include fever, cytopenias, hepatosplenomegaly, elevated ferritin, elevated triglycerides, hypofibrinogenemia, evidence of hemophagocytosis, and low NK cell activity [6]. In this case, confirmation was achieved through bone marrow biopsy, which showed hemophagocytosis.

Extreme hyperferritinemia (above 10,000 ng/mL) is a sensitive marker of severe macrophage activation, providing greater specificity for the diagnosis, although it is not exclusive [7,8]. In this patient, ferritin reached a staggering 18,366 ng/mL, a value that signals intense immune dysregulation [8].

Infections are common triggers for HLH, especially viruses such as EBV and CMV; among bacterial causes, tuberculosis is significant, particularly in regions with high prevalence and in immunosuppressed individuals. The positive urinary LF-LAM provided a diagnostic basis for extrapulmonary tuberculosis in a PLWH and allowed for the early initiation of antituberculosis therapy [9,10].

The coexistence of active infection and HLH imposes therapeutic dilemmas: immunosuppression may worsen the infection, while its absence allows the immunological storm to persist. Therefore, management requires synchrony between infection control and modulation of the immune response.

A significant recent study (2024) on tuberculosis-associated HLH indicated that the combination of antituberculosis treatment (ATT) with HLH-specific therapies, especially intravenous immunoglobulin (IVIG), significantly reduced mortality (39% overall) compared to ATT alone. This reinforces the urgency of an integrated and aggressive protocol [11-14].

In the case in question, despite combination therapy (ATT + immunosuppression), the patient progressed to multiple organ failure and death, exemplifying the aggressiveness of the condition when diagnosed late. Furthermore, the implementation of rapid tests such as LF-LAM in high TB/HIV burden settings can reduce mortality, with results available in less than 15 minutes, which is especially useful in hospital environments [6,11-14].

## Conclusions

This case report highlights the diagnostic complexity of hemophagocytic lymphohistiocytosis (HLH) in people living with HIV and low CD4 T-cell counts, where the overlap of symptoms with other severe conditions and rapid progression to multi-organ failure result in high mortality rates. The aggressive nature of the syndrome, even when immunosuppressive and antimicrobial therapies are initiated, reinforces the urgent need for a high index of clinical suspicion and the implementation of rapid diagnostic protocols, integrating sensitive methods such as the LF-LAM test, especially in tuberculosis-endemic settings. Ultimately, the case emphasizes the necessity of ongoing medical education and integrated therapeutic strategies to reduce the lethality associated with this rare and severe hyperinflammatory syndrome in vulnerable populations.

## References

[1] Ramos-Casals M, Brito-Zerón P, López-Guillermo A (2014). Adult haemophagocytic syndrome. Lancet.

[2] Knaak C, Nyvlt P, Schuster FS Hemophagocytic lymphohistiocytosis in adults: collaborative analysis of 137 cases of a nationwide German registry. J Intern Med.

[3] West J, Card TR, Bishton MJ (2022). Incidence and survival of haemophagocytic lymphohistiocytosis: A population-based cohort study from England. J Intern Med.

[4] Doyle T, Bhagani S, Cwynarski K (2009). Haemophagocytic syndrome and HIV. Curr Opin Infect Dis.

[5] Suzuki N, Morimoto A, Ohga S (2009). Characteristics of hemophagocytic lymphohistiocytosis in neonates: a nationwide survey in Japan. J Pediatr.

[6] (2025). WHO: 2.2 TB diagnosis. https://tbksp.who.int/en/node/2804.

[7] Ferreira DG, do Val Rezende P, Murao M (2014). Hemophagocytic lymphohistiocytosis: a case series of a Brazilian institution. Rev Bras Hematol Hemoter.

[8] Birndt S, Schenk T, Heinevetter B (2020). Hemophagocytic lymphohistiocytosis in adults: collaborative analysis of 137 cases of a nationwide German registry. J Cancer Res Clin Oncol.

[9] Tabaja H, Kanj A, El Zein S (2022). A review of hemophagocytic lymphohistiocytosis in patients with HIV. Open Forum Infect Dis.

[10] McKinnon AE (2024). Hemophagocytic lymphohistiocytosis secondary to miliary tuberculosis in a resource-limited setting: a case report. Cureus.

[11] Henter JI, Horne A, Aricó M (2007). HLH-2004: Diagnostic and therapeutic guidelines for hemophagocytic lymphohistiocytosis. Pediatr Blood Cancer.

[12] Liu D, Gu L, Zhang R (2022). Utility of urine lipoarabinomannan (LAM) in diagnosing mycobacteria infection among hospitalized HIV-positive patients. Int J Infect Dis.

[13] Drain PK, Niu X, Shapiro AE (2024). Real-world diagnostic accuracy of lipoarabinomannan in three non-sputum biospecimens for pulmonary tuberculosis disease. EBioMedicine.

[14] Eslami A, Alimoghadam S, Khodadadi S (2024). Comprehensive insights into tuberculosis-associated hemophagocytic lymphohistiocytosis: a systematic review. BMC Infect Dis.

